# Safety of percutaneous aortic valve insertion. A systematic review

**DOI:** 10.1186/1471-2261-9-45

**Published:** 2009-09-01

**Authors:** Hans Van Brabandt, Mattias Neyt

**Affiliations:** 1 Belgian Health Care Knowledge Centre, Administratief Centrum Kruidtuin, Kruidtuinlaan 55, 1000 Brussels, Belgium

## Abstract

**Background:**

The technique of percutaneous aortic valve implantation (PAVI) for the treatment of severe aortic stenosis (AS) has been introduced in 2002. Since then, many thousands such devices have worldwide been implanted in patients at high risk for conventional surgery. The procedure related mortality associated with PAVI as reported in published case series is substantial, although the intervention has never been formally compared with standard surgery. The objective of this study was to assess the safety of PAVI, and to compare it with published data reporting the risk associated with conventional aortic valve replacement in high-risk subjects.

**Methods:**

Studies published in peer reviewed journals and presented at international meetings were searched in major medical databases. Further data were obtained from dedicated websites and through contacts with manufacturers. The following data were extracted: patient characteristics, success rate of valve insertion, operative risk status, early and late all-cause mortality.

**Results:**

The first PAVI has been performed in 2002. Because of procedural complexity, the original transvenous approach from 2004 on has been replaced by the transarterial and transapical routes. Data originating from nearly 2700 non-transvenous PAVIs were identified. In order to reduce the impact of technical refinements and the procedural learning curve, procedure related safety data from series starting recruitment in April 2007 or later (n = 1975) were focused on. One-month mortality rates range from 6.4 to 7.4% in transfemoral (TF) and 11.6 to 18.6% in transapical (TA) series. Observational data from surgical series in patients with a comparable predicted operative risk, indicate mortality rates that are similar to those in TF PAVI but substantially lower than in TA PAVI. From all identified PAVI series, 6-month mortality rates, reflecting both procedural risk and mortality related to underlying co-morbidities, range from 10.0-25.0% in TF and 26.1-42.8% in TA series. It is not known what the survival of these patients would have been, had they been treated medically or by conventional surgery.

**Conclusion:**

Safety issues and short-term survival represent a major drawback for the implementation of PAVI, especially for the TA approach. Results from an ongoing randomised controlled trial (RCT) should be awaited before further using this technique in routine clinical practice. In the meantime, both for safety concerns and for ethical reasons, patients should only be subjected to PAVI within the boundaries of such an RCT.

## Background

The first human percutaneous aortic valve implantation (PAVI) has been performed by Cribier in April 2002[1]. The initial antegrade transvenous technique through the interatrial septum later on was replaced by the retrograde transarterial approach. For patients in whom vascular access was rendered impossible due to severe atheromatous disease, the transapical route was designed, where PAVI occurs directly through the left ventricular apex, involving a mini-thoracotomy. So far, results from over 3500 PAVIs have been made public[2,3]. Although both European[4] and American[5] professional organisations emphasise safety issues, many single or multiple case series continue to be reported. We recently performed a Health Technology Assessment on PAVI[6]. The safety issues emerging from this review are discussed here.

## Methods

On December 15, 2008, both authors searched Medline, Embase, Cochrane Library, and CRD databases, using relevant subject headings and a collection of text words representing the concept of PAVI. Furthermore, internet-based sources dedicated to dissemination of results from cardiovascular trials ,  were searched. We also contacted interventional cardiologists and manufacturers. Full details of the search strategy have been described elsewhere[6] and are accessible from our website . To be eligible for inclusion, studies had to report experience with transarterial or transapical PAVI in patients with severe symptomatic aortic stenosis (AS). Transvenous PAVI was not considered. Single-case reports were excluded because of their obvious anecdotal nature. Only devices that have been granted European CE marking were taken into consideration: the Edwards Lifesciences device (or any of its predecessors) and the CoreValve Revalving System. The CE marking denotes a formal statement by the manufacturer of compliance with EU directives requirements. Unlike the pharmaceutical sector, where new drugs have to undergo series of regulatory clinical trials during development, the evaluation of medical devices is less demarcated and no pre-market clinical trials are required for obtaining CE marking[7].

The following data were extracted: operative risk score, valve type and approach, number of patients in the series, time frame of the series, implantation success rate, procedural (defined as 30-day) mortality, and longer term mortality if available.

## Results

CRD and the Cochrane database revealed no systematic reviews on PAVI. No data from randomised controlled trials (RCTs) were identified. The National Institutes of Health clinical trials registry  mentioned one RCT in progress. Our selection process resulted in 15 case series that further in this review are referred to as "published series". Extracted data are depicted in Table 1.

**Table 1:** Data extracted from "published series"

**Reference**	**Time window**	**n**	**Age in years mean ± SD (median: IQR)**	**Logistic EuroSCORE mean ± SD (median: IQR)**	**STS score**	**Device, approach**	**Success rate (%)**	**30-d mort (%)**	**6-m mort (%)**
[27]	Jan 2005 - July 2005	18	81 ± 6	26.2 ± 13.1	NA	Ed, TF	77.8	11.1	NA

[23]	Jan 2005 - NA	50	82 ± 7	28	NA	Ed, TF	86	12	18

[28]	Feb 2005 - Nov 2005	25	80.3 ± 5.4	(10.97:19.90-9.20)	NA	CV, TF	84	20	NA

[29]	March 2005 - Aug 2006	11	81.8 ± 6.8	(36: 5-48)	NA	CV, TF	100	18.2	NA

[30]	Aug 2005 - Feb 2007	86	82.2 ± 5.9	21.7 ± 12.6	NA	CV, TF	88	12	NA

[31]	Oct 2005 - NA	7	63-91	range: 7-66	NA	Ed, TA	100	14.3	42.8

[32]§	Oct 2005 - NA	7	63-91	range: 7-66	NA	Ed, TA	§	§	§

[33]	Dec 2005 - Aug 2006	10	(81.3: 64-85)	(32: 21-40)	NA	CV, TF	100	20	NA

[34]	Feb 2006 - Sep 2006	30	82 ± 5	27.1 ± 12.2	NA	Ed, TA	96.6	6.6	NA

[35]	Feb 2006 - March 2007	50	82.4 ± 4.6	276 ± 12.2	15.8 ± 9.1	Ed, TA	94	8	26.1

[21]	Feb 2006 - Oct 2006	59	81.4 ± 5.8	26.8 ± 13.5	NA	Ed, TA	93.2	13.6	NA

[36]	Feb 2006 - Feb 2008	26	84 ± 7	36.5 ± 5.8	NA	Ed, TA	100	15	NA

[37]	Oct 2006 - Apr 2007	12	85 ± 6	31.1 ± 14.4	18.8 ± 4.3	Ed, TF	83	25	25

[14]	Dec 2006 - Feb 2008	40	83 ± 7.5	35.5 ± 15.3	13.4	Ed, TA	87.5	17.5	41.3

[38]	April 2007 - January 2008	22	84 ± 7	26 ± 16*		Ed, TF+TA	91	9.1	NA

NA: not available. Ed: Edwards Lifesciences device (or any of its predecessors). CV: CoreValve Revalving System. TF: transfemoral. TA: transapical. § 6-month follow-up of data from [31] *: risk estimation tool not mentioned. SD: standard deviation. IRQ: interquartile range. NA: not available. Success: % of attempted successful. STS: Society of Thoracic Surgeons.

Unpublished data were obtained through contacts with manufacturers and from webposted 2008 conference proceedings: EuroPCR08, Transcatheter Valve Therapy, European Association for Cardio-Thoracic Surgery and Transcatheter Cardiovascular Therapeutics. These unpublished data are referred to as "presented series" further in this review. Thirteen case series (Table 2) with a dedicated acronym were identified; reference to these in the published series rarely occurred. Based on the names of participating authors and on the time window of patient recruitment, it seems that most of the cases that have been discussed in published papers are part of the presented series. Patient characteristics and outcome data from presented series are provided in Table 2. Data from 2692 patients were identified, 76% of which were treated via the transfemoral (TF) and 24% via the transapical (TA) route. Their mean age was about 82 years. In all series operative risk was estimated by means of the logistic EuroSCORE . Some series did also provide the risk by the Society of Thoracic Surgeons (STS) risk tool . The EuroSCORE of TF treated patients was lower than that of TA treated subjects. Data related to the same registry were not always identical from different sources. In Table 2, we entered the most recently presented data that we could identify. Technical success of PAVI was not always defined in the same way among different registries. For example, in a report of the PARTNER EU registry, Lefèvre reports a 96.3% success rate (52/54), although PAVI was reportedly aborted in 6 patients out of 60 planned because of vascular access problems, a failed balloon valvuloplasty or the detection of endocarditis[8] Incorporating these data would result in a success rate of 86.7% (52/60).

**Table 2:** Data extracted from "presented series"

**Registry acronym**	**Time window**	**n**	**Age**	**Logistic EuroSCORE**	**STS score**	**Success rate (%)**	**30-d mort**	**6-m mort**	**1-yr mort**	**Data source**
Transfemoral Edwards Lifesciences PAVI										

VANCOUVER	Jan 2005 - NA	114	84°	28°	9.1°	92.1	7.9	13**	20**	[39]

REVIVAL 2	Dec 2005 - Feb 2008	55	82.8 ± 6.8	34.1 ± 18.0	13.1 ± 7.2	87.3	7.3	16.6	24.2	[40,41]

REVIVE 2	NA - Dec 2007	106	83.9 ± 5.4	29.9 ± 13.2	NA	88.0	13.2	21.4	28.6	[40,41]

PARTNER EU	April 2007 - Jan 2008	60	82.5 ± 5.2	24.7 ± 11.7	10.9 ± 5.9	96.3	7.4	10	NA	[8]

SOURCE	Nov 2007 - Sep 2008	293	81.8	26.4	NA	97.7	6.4	NA	NA	[8]

Transfemoral CoreValve PAVI										

CoreValve 21F S&E	Aug 2005 - 2006	52	81.4 ± 5.5	27.4 ± 15.1	NA	90.4	15.4	23**	35**	[2]

CoreValve 18F S&E	May 2006 - Oct 2007	124	81.8 ± 6.5	23.0 ± 13.5	NA	94.4	14.5	23**	28**	[2]

CoreValve 18F EE	April 2007 - Sep 2008	1243	81.2 ± 6.4	22.9 ± 14.1	NA	98.2	6.7	21°	NA	[2,40]

**TOTAL TRANSFEMORAL**		**2047**					**7.8**			

Transapical Edwards Lifesciences PAVI										

VANCOUVER	Oct 2005 - NA	58	84°	28°	9.1°	98.3	19.0	34	34	[39]

REVIVAL 2	Dec 2005 - Feb 2008	40	83.7 ± 5.2	35.5 ± 15.3	13.4 ± 7.0	87.5	17.6	35.8	45.3	[3,14]

TRAVERCE	Dec 2004 - April 2008	168	82.0 ± 5.6	26.9 ± 12.8	NA	92.9	14.9	30	35	[41]

PARTNER EU	April 2007 - Jan 2008	70	82.1 ± 5.7	33.5 ± 14.8	11.9 ± 7.0	91	18.6	42	NA	[8]

SOURCE	Nov 2007 - Sep 2008	309	80.7	30	NA	NA	11.6	NA	NA	[42]

**TOTAL TRANSAPICAL**		**645**					**13.8**			

Registry acronyms: VANCOUVER: Vancouver single-centre experience; REVIVAL 2: peRcutaneous EndoVascular Implantation of VALves; REVIVE 2: Registry of EndoVascular Implantation of Valves in Europe; PARTNER EU: Placement of AoRTic TraNscathetER Valve Trial; SOURCE: edwards Sapien aOrtic bioprothesis eURropean outComE registry; CoreValve 21F S&E: 21 French safety and efficacy registry; CoreValve 18F S&E: 18 French safety and efficacy registry; CoreValve 18F EE: 18 French post CE mark Expanded Evaluation registry; TRAVERCE: TRAnsapical Surgical DeliVEry of the Cribier-Edwards aortic bioprosthesis. TF: transfemoral. TA: transapical. PAVI: percutaneous aortic valve insertion. *: 18 French and 21 French refer to the outer diameter of the delivery sheath. **: estimated from Kaplan Meier survival plot. °: data from TF and TA series combined.

In order to reduce the impact of technical refinements and the procedural learning curve, a summary estimate of PAVI safety is calculated from series starting recruitment in April 2007 or later (n = 1975). In these registries, one-month mortality rates range from 6.4 to 7.4% in TF and 11.6 to 18.6% in TA series. From all identified PAVI series, six-month mortality ranges from 10-25.0% following TF and 26.1-42.8% following TA procedures.

## Discussion

By introducing a percutaneous approach for aortic valve insertion, it was hypothesised that elderly and frail patients in whom this less-invasive procedure was performed would run a lower procedural risk than when treated by conventional aortic valve replacement (AVR)[9]. Since the first PAVI in 2002, many thousands such devices have been implanted worldwide. In the initial antegrade transseptal approach a procedure attributable mortality risk of 25% was noted,[10] which initiated the development of the less demanding retrograde TF technique. The latter can however be hampered due to difficulties to advance large catheters through tortuous and diffusely diseased femoral and iliac arteries, often encountered in elderly people. These restrictions have led to the development of the TA approach where the same device is introduced from the cardiac apex via a mini-thoracotomy. Due to this selection process, patients treated by the TA route mostly have a higher risk profile than those treated by the TF approach which is reflected in their higher EuroSCORE.

The available data demonstrate that even at experienced centers, PAVI remains a risky procedure. In series starting recruitment in April 2007 or later, i.e. after European market approval, one-month mortality rates range from 6.4 to 7.4% in TF and 11.6 to 18.6% in TA series. In these patients, it is uncertain what their survival would have been if they had been operated conventionally or treated medically. It also remains unclear to what extent a patient's overall quality of life is improved by the procedure, provided he or she survives the intervention. There are no sound criteria to assess the appropriateness to proceed to correction of a symptomatic AS (whether by surgery or PAVI) in frail elderly patients with substantial co-morbidities. Depending on the pre-procedural clinical condition that remains unaffected by correcting the AS, the quality of life may hardly be altered by a successful PAVI. In this respect, recent guidelines state that "valve replacement is technically possible at any age, but the decision to proceed with such surgery depends on many factors ... Deconditioned and debilitated patients often do not return to an active existence, and the presence of the other comorbid disorders could have a major impact on outcome[11]."

In a literature review on AVR in the octogenarian, operative mortality of isolated AVR varied between 4.3 and 10.3%[12]. Very recently operative results of 1000 "minimally invasive" (i.e. parasternal approach or hemisternotomy) AVRs were reported. Among 160 patients of 80 years or older undergoing isolated AVR, operative mortality rate was 1.9%[13]. Svensson et al. report the fate of 163 patients that were referred to their institutions for potential PAVI because of putative inoperability[14]. Twenty nine of them were treated by conventional AVR with no operative deaths. High-surgical-risk and operability status are poorly defined concepts and complicate the interpretation of outcomes from observational data. The operative risk of patients contemplated for PAVI have mostly been estimated through the EuroSCORE but its performance in high-risk patients has been critisised [15-19]. Recent observational data from surgically treated patients indicate that the EuroSCORE severely overestimates postoperative mortality in high-risk patients undergoing an isolated AVR. In a surgical series from the Mayo Clinic, a predicted 30-day mortality of 23.6% sharply contrasted with an observed mortality of only 5.8%[20]. By comparing this figure with 30-day mortality rates observed in PAVI (Table 2), it could be argued that patients with AS that are considered at high risk for conventional surgery, may actually run a higher mortality risk if treated by means of PAVI than when treated surgically. Only data from an RCT would enable to clarify this.

Six-month mortality of patients treated by PAVI, reflecting both procedural risk and mortality from underlying co-morbidities, is also very high and ranges from 10-25.0% in TF series and 26.1-42.8% in TA series (Tables 1 and 2). The wide range in the observed short term mortality rate may result from the chosen interventional approach for PAVI and from differences in patient selection. The EuroSCORE does not take into account several conditions that are often encountered in elderly patients, yet are determinants for both life expectancy and quality of life, even following successful PAVI: coronary artery disease, heart failure, diabetes, presence and degree of mitral regurgitation, arrhythmias, previous stroke, renal failure on dialysis[21,22]. This might explain that in a series of 50 patients reported by Webb, 6-month survival of 7 patients in whom PAVI failed (86%) was similar to 6-month survival of 43 successfully treated cases (81%)[23].

One-year survival after PAVI ranges from 65-80% in TF series and from 54.7-66% in TA series. In an observational study encompassing 277 elderly patients (> 80 years) 80 underwent surgical AVR and 197 were treated medically. One-year survival among patients with AVR was 87%, compared with 52% in those who had no AVR[24]. Data from the European Heart Survey showed a 1-year survival among 72 patients (> 75 years old) with severe symptomatic AS in whom it was decided not to operate, of 84.8 ± 4.8%[25]. In a US series of 75 unoperated patients aged 68.1 ± 15.0 years, with severe symptomatic AS, one-year survival was 62%[26]. The poor one-year survival of patients following PAVI overlaps with that of patients treated conservatively. This can at least partly be explained by a high procedural mortality rate and the very poor general condition with inherent competing mortality risk of the patients involved, questioning the appropriateness of an intervention directed towards a correction of the AS in this population.

The major shortcoming of published series and cases presented at international meetings, the data of which are summarised here, is the lack of randomisation of eligible patients to an intervention group treated with PAVI, versus a control group. Moreover, data published in peer reviewed journals reflect the experience of specified authors, whereas the data from compiled PAVI series presented at meetings are entirely under control of the manufacturers involved. Participation in some of the registries and the reporting of outcomes in some instances is on a voluntary basis only. We were unable to verify the completeness and the correctness of reported mortality rates. One-year mortality rates that are referred to in this paper obviously stem from earlier series, and may not be applicable to more recent experience. Therefore, the results of our study and from any non-randomised trial should be interpreted with caution.

Although data from an increasing number of patients are published in journals and presented at meetings, they do not add to our understanding of the potential of this new technology. The methodological shortcomings prevailing in all these series remain unaltered, i.e. the data are merely observational from selected and unrandomised patient groups.

## Conclusion

The safety issues pointed out above reinforce the contention that an RCT is badly needed to clarify the safety and the performance of PAVI in frail and elderly patients. In the years to come, evidence will be provided by an ongoing RCT, the PARTNER-IDE (ClinicalTrials.gov identifier NCT00530894)[3]. One might wonder whether in the meantime subjecting patients to PAVI can be justified.

## Competing interests

Both authors are employees of KCE , an independent semi-governmental Health Technology Assessment agency and member of INAHTA . We have no ties with industry or any other commercial organisation. We have no conflicts of interest.

## Authors' contributions

Both authors contributed equally to this work.

## Pre-publication history

The pre-publication history for this paper can be accessed here:


